# Evaluating Large Language Models in Dental Anesthesiology: A Comparative Analysis of ChatGPT-4, Claude 3 Opus, and Gemini 1.0 on the Japanese Dental Society of Anesthesiology Board Certification Exam

**DOI:** 10.7759/cureus.70302

**Published:** 2024-09-27

**Authors:** Misaki Fujimoto, Hidetaka Kuroda, Tomomi Katayama, Atsuki Yamaguchi, Norika Katagiri, Keita Kagawa, Shota Tsukimoto, Akito Nakano, Uno Imaizumi, Aiji Sato-Boku, Naotaka Kishimoto, Tomoki Itamiya, Kanta Kido, Takuro Sanuki

**Affiliations:** 1 Department of Dental Anesthesiology, Kanagawa Dental University, Yokosuka, JPN; 2 Department of Liberal Arts Education, Kanagawa Dental University, Yokosuka, JPN; 3 Department of Anesthesiology, Aichi Gakuin University, Nagoya, JPN; 4 Division of Dental Anesthesiology, Faculty of Dentistry and Graduate School of Medical and Dental Sciences, Niigata University, Niigata, JPN; 5 Department of Dental Anesthesiology, Hokkaido University, Sapporo, JPN

**Keywords:** chatgpt, claude, dental anesthesiology, gemini, generative artificial intelligence, large language models

## Abstract

Purpose

Large language models (LLMs) are increasingly employed across various fields, including medicine and dentistry. In the field of dental anesthesiology, LLM is expected to enhance the efficiency of information gathering, patient outcomes, and education. This study evaluates the performance of different LLMs in answering questions from the Japanese Dental Society of Anesthesiology Board Certification Examination (JDSABCE) to determine their utility in dental anesthesiology.

Methods

The study assessed three LLMs, ChatGPT-4 (OpenAI, San Francisco, California, United States), Gemini 1.0 (Google, Mountain View, California, United States), and Claude 3 Opus (Anthropic, San Francisco, California, United States), using multiple-choice questions from the 2020 to 2022 JDSABCE exams. Each LLM answered these questions three times. The study excluded questions involving figures or deemed inappropriate. The primary outcome was the accuracy rate of each LLM, with secondary analysis focusing on six subgroups: (1) basic physiology necessary for general anesthesia, (2) local anesthesia, (3) sedation and general anesthesia, (4) diseases and patient management methods that pose challenges in systemic management, (5) pain management, and (6) shock and cardiopulmonary resuscitation. Statistical analysis was performed using one-way ANOVA with Dunnett's multiple comparisons, with a significance threshold of p<0.05.

Results

ChatGPT-4 achieved a correct answer rate of 51.2% (95% CI: 42.78-60.56, p=0.003) and Claude 3 Opus 47.4% (95% CI: 43.45-51.44, p<0.001), both significantly higher than Gemini 1.0, which had a rate of 30.3% (95% CI: 26.53-34.14). In subgroup analyses, ChatGPT-4 and Claude 3 Opus demonstrated superior performance in basic physiology, sedation and general anesthesia, and systemic management challenges compared to Gemini 1.0. Notably, ChatGPT-4 excelled in questions related to systemic management (62.5%) and Claude 3 Opus in pain management (61.53%).

Conclusions

ChatGPT-4 and Claude 3 Opus exhibit potential for use in dental anesthesiology, outperforming Gemini 1.0. However, their current accuracy rates are insufficient for reliable clinical use. These findings have significant implications for dental anesthesiology practice and education, including educational support, clinical decision support, and continuing education. To enhance LLM utility in dental anesthesiology, it is crucial to increase the availability of high-quality information online and refine prompt engineering to better guide LLM responses.

## Introduction

Large language models (LLMs) are a type of generative artificial intelligence that learns from vast amounts of textual data sourced from internet articles, books, and web pages to create artificial intelligence models for natural language processing (NLP). As society becomes more information-oriented, patients increasingly have opportunities to gather information about diseases, treatments, and medical institutions from sources other than healthcare providers. According to a survey by the Ministry of Health, Labor and Welfare, 23.5% of patients use the internet as a tool to obtain information when visiting a medical institution [1]. A similar trend has been observed in student education. According to a 2013 survey by the Ministry of Internal Affairs and Communications' Institute for Information and Communications Policy, about 97% of university students used the internet for information searches. While internet searches provide easy access to large amounts of information, there are concerns about potential confusion and anxiety due to the insufficient verification of consistency between information sources [2].

The use of LLMs is advancing in various fields, including medicine. A study that had ChatGPT, an LLM, take the United States Medical Licensing Examination (USMLE) reported achieving scores at or near passing standards without special reinforcement learning or fine-tuning, suggesting the potential for LLMs to support medical education and clinical decision-making [3]. LLMs generate words and sentences based on probability distributions using web information as learning data. Due to this probabilistic nature, different responses may be generated when the same question is asked multiple times to the same LLM or to different LLMs. A study that had ChatGPT answer the Japanese medical licensing examination reported that the accuracy rates for each medical field significantly correlated with the number of papers in that field, with lower accuracy for topics with less information, such as new drugs and diseases [4]. Therefore, there may be room for debate regarding the appropriate use of LLMs.

In dentistry, potential applications of LLMs are being discussed in areas such as teledentistry, clinical decision support, administrative efficiency, patient education, and dental school education [5]. The field of dental anesthesiology, which deals with general anesthesia, sedation, and pain management in dentistry, also holds promise for LLM utilization. The potential benefits of implementing LLMs include improved information-gathering efficiency, patient outcomes, and dental anesthesiology education, possibly leading to safer dental care for more patients. Therefore, this study aims to evaluate which LLMs could potentially function usefully in dental anesthesiology by comparing multiple LLMs.

## Materials and methods

Study design

The study focused on the Japanese Dental Society of Anesthesiology Board Certification Examination (JDSABCE) from 2020 to 2022 (45th to 47th sessions). Three different LLMs were tasked with answering the exam questions three times each. Each JDSABCE consists of five written questions and 100 multiple-choice questions (MCQs). The study included only the MCQs. The exam contained two types of questions: those requiring the selection of 1-3 correct answers from five options and those requiring the selection of all correct answers from the given options. The original JDSABCE questions were presented in Japanese and manually input one by one into the chat interfaces of non-fine-tuned LLMs. No special prompt engineering was employed.

The LLMs used were ChatGPT-4 (OpenAI, San Francisco, California, United States), Gemini 1.0 (Google, Mountain View, California, United States), and Claude 3 Opus (Anthropic, San Francisco, California, United States). Gemini 1.0 was freely available, while ChatGPT-4 and Claude 3 Opus were high-performance models accessible through monthly subscriptions. The LLMs used in this study did not have image recognition capabilities. Therefore, questions containing figures or tables and those deemed inappropriate by society were excluded. The primary outcome measure was the accuracy rate of each LLM. The JDSABCE questions were input into the three LLMs over a two-week period from March 14 to 30, 2024. As the correct answers to the exam questions were not publicly available, answers were considered correct when they matched answers based on discussions among multiple board-certified dental anesthesiologists (HK, KK, NK, ST, UI, AS, NK, KK, and TS).

Subspecialty-specific evaluation

The MCQs in the JDSABCE are designed to test the fundamental knowledge and skills required of dental anesthesiology specialists. The exam covers not only basic medical sciences such as physiology and pharmacology related to general anesthesia and pain management but also clinical medicine based on various diagnostic and treatment guidelines. Therefore, the subject areas were subdivided into six subgroups: (1) basic physiology necessary for general anesthesia, (2) local anesthesia, (3) sedation and general anesthesia, (4) diseases and patient management methods that pose challenges in systemic management, (5) pain management, and (6) shock and cardiopulmonary resuscitation. The percentage of correct answers for each subgroup was set as a secondary outcome measure. The MCQs from the JDSABCE were independently reviewed and categorized by four dental anesthesiologists (MF, NK, TK, and HK). Each question was assigned to one of the six subgroups based on its primary focus and the core competency it assessed. In cases of disagreement, the final categorization was determined by consensus among the dental anesthesiologists. The six subgroups were chosen to reflect the key areas of knowledge and competence required in dental anesthesiology, as outlined in the current dental anesthesiology curricula.

Statistical analysis

Data are expressed as the mean±standard deviation (SD) or 95% confidence interval (CI) of 295 questions, where n represents the percentage of correct answers given three times. Statistical significance was determined using one-way ANOVA with Dunnett's multiple comparisons. The threshold for statistical significance was set at p<0.05. All statistical analyses were performed using GraphPad Prism 7.05 (GraphPad Software, La Jolla, California, United States).

Ethical considerations

This study was conducted at Kanagawa Dental University and used only publicly available data from the internet. It did not involve human or animal subjects. Therefore, specific ethical considerations were exempted.

## Results

Comparison of percentages of correct answers for JDSABCE among three different LLMs

**Figure 1:**
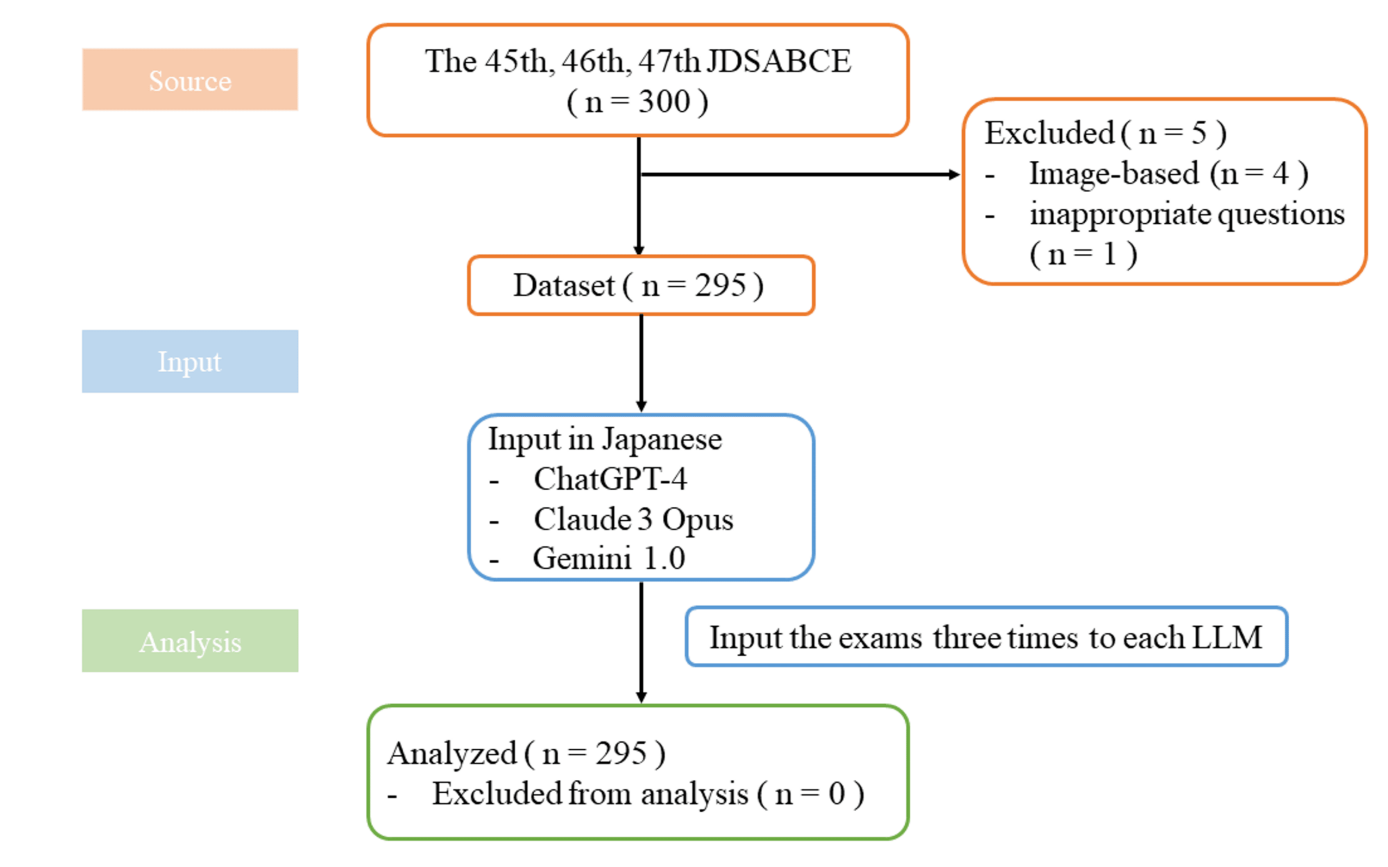
Flow diagram of study design and setting A total of 295 questions were included in the study, and all results were incorporated into the analysis. JDSABCE: Japanese Dental Society of Anesthesiology Board Certification Examination

The 45th to 47th JDSABCE each contained 100 questions. Of these, four questions included figures or tables, and one question was deemed inappropriate by the society. Consequently, a total of 295 questions (99 from the 45th, 98 from the 46th, and 98 from the 47th exams) were included in the study (Figure 1).

**Figure 2:**
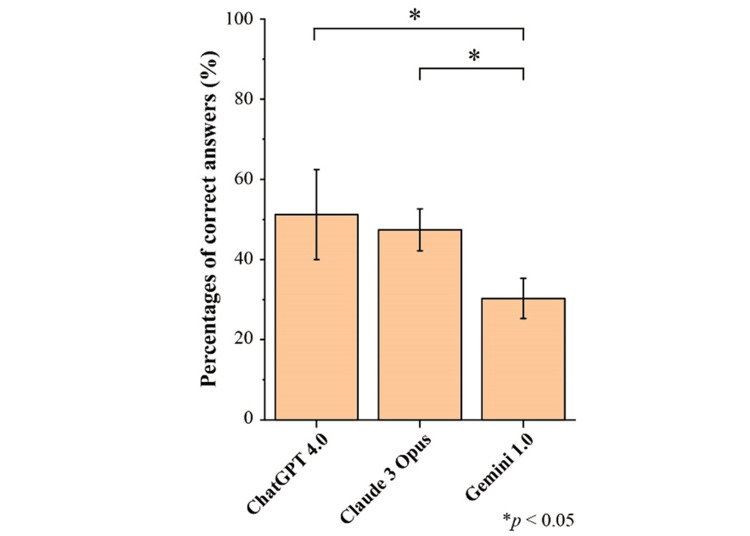
The percentages of correct answers for the JDSABCE Data are expressed as the mean±SD of 295 questions, which represents the percentage of correct answers given three times. Significant differences are indicated using asterisks. *: p<0.05; JDSABCE: Japanese Dental Society of Anesthesiology Board Certification Examination; SD: standard deviation

The percentages of correct answers for the JDSABCE were significantly higher for ChatGPT-4 (51.2±11.2%, 95% CI: 42.78-60.56, p=0.003) and Claude 3 Opus (47.4±5.2%, 95% CI: 43.45-51.44, p<0.001) compared to Gemini 1.0 (30.3±5.0%, 95% CI: 26.53-34.14) (Figure 2).

Comparison of percentages of correct answers by subject area

To investigate factors influencing the higher accuracy rates of ChatGPT-4 and Claude 3 Opus, a subgroup analysis was conducted for each subject area. The percentages of correct answers for each subject area are shown in Table 1.

**Table 1:** Percentage of correct answers for each LLM in subgroups (%) Data are expressed as the mean±95% CI. Significant differences are indicated using asterisks or tagger. *: p<0.05, ChatGPT-4 vs Gemini 1.0. **:p<0.05, Claude 3 Opus vs Gemini 1.0. †: p<0.05, ChatGPT-4 vs Claude 3 Opus; CI: confidence interval; LLM: large language model

Subject area	ChatGPT-4 (95% CI)	Claude３Opus (95% CI)	Gemini 1.0 (95% CI)	Statistical significance
Basic physiology for systemic management	49.49 (34.67-64.31)	57.59 (46.77-68.4)	33.17 (21.16-45.17)	*, **
Local anesthesia	30.93 (17.43-44.43)	36.56 (25.52-47.59)	23.3 (10.76-35.84)	
Sedation and general anesthesia	50.4 (38.99-61.81)	44.47 (38.84-50.1)	25.98 (21.68-30.27)	*, **
Diseases that cause systemic management problems and management methods	62.5 (47.16-77.84)	49.61 (43.98-55.24)	39.13 (32.01-46.26)	*, **
Pain management	49.33 (34.48-64.19)	61.53 (50.44-72.63)	27.24 (14.51-39.98)	**
Shock and cardiopulmonary resuscitation	43.61 (29.36-57.87)	24.17 (14.7-33.63)	15.56 (-2.921-34.03)	*, †

ChatGPT-4 and Claude 3 Opus demonstrated significantly higher percentages of correct answers compared to Gemini 1.0 in three areas as follows: (1) basic physiology necessary for systemic management, (2) sedation and general anesthesia, and (3) diseases and patient management methods that pose challenges in systemic management. Notably, percentages of correct answers exceeding 60% were observed in two instances as follows: (1) ChatGPT-4 performance on questions related to diseases and patient management methods that pose challenges in systemic management (62.5%, 95% CI: 47.16-77.84) and (2) Claude 3 Opus performance on questions related to pain management (61.53%, 95% CI: 50.44-72.63).

## Discussion

In this study, the percentages of correct answers for ChatGPT-4, Claude 3 Opus, and Gemini 1.0 to the 45th through 47th JDSABCE were approximately 30-60%, slightly lower for Gemini 1.0 compared to ChatGPT-4 and Claude 3 Opus. This result was influenced by the percentages of correct answers in three categories: basic physiology for systemic management, sedation and general anesthesia, and diseases that cause systemic management problems and management methods.

Several studies, both national and international, have shown high rates of correct responses by LLMs on medical licensing examinations. A study on the 117th Japanese Medical Licensing Examination (JMLE) reported that ChatGPT-4 achieved correct answer rates of 87.2% for essential knowledge questions, 73.3% for general clinical questions, and 81.7% for specific disease questions. These scores met the passing rates for the 117th JMLE [6]. Another study evaluated multiple LLMs on the 2023 Peruvian National Licensing Medical Examination. ChatGPT-4 and Bing achieved correct answer rates exceeding 80%, while ChatGPT-3, Bard, and Claude scored approximately 60% [7]. Regarding the United States Medical Licensing Examination (USMLE), a report showed that ChatGPT's performance exceeded 60% correct answers in both Step 1 and Step 2 areas. This performance was comparable to that expected of a third-year medical student in the United States [8]. Previous studies have shown high rates of correct responses by LLMs on various medical licensing examinations. However, in this study, all LLMs achieved correct response rates below 60% on the JDSABCE. There were three possible reasons for these results: (1) limited information about dental anesthesiology on the web, (2) lack of prompt engineering specific to this exam, and (3) ambiguity in language and question content of the questions.

In previous reports, many answers and explanations to medical exam questions were readily available on the web. However, for the JDSABCE, there exists a closed, subscription-based online community with exam commentaries, but this information is not freely accessible elsewhere. This limited availability of dental anesthesiology information on the web may explain the lower performance of LLMs compared to previous reports. The subgroup analysis in this study revealed relatively high percentages of correct answers for questions categorized as diseases that cause systemic management problems and pain management. These results suggest that information on general pathophysiology and common medical management is more widely available online. In contrast, specialized information on local and general anesthesia, as well as sedation techniques specific to dental procedures, may be more limited on the web.

Another possible reason for the low percentages of correct answers by LLMs in this study was suboptimal prompt engineering. Artificial intelligence prompts are directives that guide LLMs to generate specific outputs. A study examining prompt tuning for JMLE showed that using the prompt "Answer the following question with a reason" and clarifying the structure before inputting the question increased the percentage of correct responses [9-11]. Similarly, a study using ChatGPT-4 to answer the 112th through 115th Japanese National Dental Examinations employed the prompt "You are a dental student taking the National Dental Examination. Please answer the questions according to the question text." in Japanese. This approach resulted in nearly 80% correct answers for essential questions, with dental anesthesiology questions achieving 100% accuracy [12]. Optimizing prompts can clarify the expected output from the LLM, potentially leading to more appropriate answers. In contrast, this study input JDSABCE questions in their original Japanese text without specific prompts, which may have affected performance. Furthermore, a study comparing English and Japanese inputs of JMLE questions to ChatGPT-4 reported a higher percentage of correct answers for English inputs than that for Japanese, even after subtracting errors caused by improper translations [9]. This study suggests that the accuracy of LLM responses may have been affected by the lack of prompts and the potential ambiguity in Japanese. This study revealed varying percentages of correct responses across different question types, indicating each LLM may have strengths and weaknesses in specific areas. These differences likely stem from variations in the training data used for each model. In order to effectively utilize LLMs in dental anesthesiology, optimization of prompt engineering as well as careful selection of LLM types should be considered.

This study has several limitations. First, the time at which we input the JDSABCE questions into the LLMs was somewhat past and did not use the most recent versions of LLMs, which are expected to evolve over time as they are learned. This temporal gap is significant because LLMs are rapidly improving, and the latest versions may potentially achieve higher accuracy rates than those observed in our study. Therefore, if we were to repeat this study now with the most up-to-date LLMs, we might see improved performance in answering the JDSABCE questions. Second, artificial hallucination is difficult for non-experts to identify: LLMs may produce statistically "plausible" answers, or hallucinations, although they contain incorrect information. Hallucinations can manifest in various ways including faulty logical reasoning, misquoting or misinterpreting web data, and calculation mistakes. Because LLM responses are based on statistical inference, increasing training data or improving prompts will not ensure that these hallucinations can be eliminated. There is no established method to reliably exclude hallucination and extract only correct responses from LLMs. Therefore, expert judgment is required to assess the accuracy of generated answers. This poses a risk that users without sufficient knowledge of dental anesthesiology, such as students and patients, may become confused when using LLMs due to their ability to generate plausible but potentially inaccurate responses. In the medical field, a qualified person makes the final decision. Therefore, it is also debatable what level of accuracy should be considered acceptable for LLM usefulness. A demonstration experiment conducted at Tohoku University Hospital showed that medical LLMs could automatically generate a summary text of the course of treatment, reducing the time required for creating referral letters and discharge summaries by an average of 47%, although it was necessary to take measures against hallucination [13]. LLMs may be more suitable for administrative tasks in dental anesthesia practice, such as summarizing existing data, rather than assisting in diagnosis or answering complex questions about pathophysiology and symptoms. Another limitation of this study is the difficulty in conducting a detailed analysis of the causes behind the low accuracy rates of LLMs. A particularly intriguing point is the low accuracy rates in areas such as shock and resuscitation, where general medical information is presumed to be abundant. This unexpected result could potentially be attributed to the following main factors: (1) difficulties in adapting to the specific context of dental anesthesiology, (2) reflection of the latest guidelines and practices, and (3) language and cultural factors specific to the Japanese dental field. However, due to the design of our study, it was not possible to analyze these factors individually and clearly establish their impact. Furthermore, we were unable to conduct a detailed analysis of how the specific characteristics of the JDSABCE questions might have influenced LLM performance compared to other medical licensing exams. Factors such as potential ambiguities unique to JDSABCE question texts or a possibly higher ratio of questions testing clinical judgment could have affected the LLMs' accuracy. However, due to the design of our study, it was not possible to perform a detailed comparative analysis between the JDSABCE and other medical licensing exams. Another limitation of this study is the decision not to use prompts in evaluating the LLMs. The use of appropriate prompts could potentially enhance the performance of LLMs. While we chose not to use prompts in this study to assess the baseline capabilities of LLMs and to maintain uniform conditions for comparison, this approach may have underestimated their potential performance. Regardless, the key feature of LLMs is their ability to generate text based on web-sourced training data. To enhance LLM's usefulness in dental anesthesiology, it is crucial to disseminate more accurate and comprehensive information about the field online.

## Conclusions

This study showed potential for useful application in dental anesthesiology of ChatGPT-4 and Claude 3 Opus compared to Gemini 1.0. However, the accuracy rates for the JDSABCE were below 60% for all LLMs, indicating that their current utility as tools in dental anesthesiology may be insufficient. Nevertheless, given the rapid advancements in LLM technology, we can anticipate improvements in accuracy in the future. For LLMs to become valuable tools in dental anesthesiology, it is crucial that we continue to disseminate appropriate information related to the field. Furthermore, we need to consider presenting this information in a format that is conducive to LLM learning, avoiding ambiguous expressions.
